# Occurrence of *Pasteurella multocida* in Dogs Being Trained for Animal-Assisted Therapy

**DOI:** 10.3390/ijerph17176385

**Published:** 2020-09-02

**Authors:** Antonio Santaniello, Susanne Garzillo, Alessia Amato, Mario Sansone, Alessandro Fioretti, Lucia Francesca Menna

**Affiliations:** 1 Department of Veterinary Medicine and Animal Productions, Federico II University of Naples, 80134 Naples, Italy; susannegarzillo@gmail.com (S.G.); alessiaamatovet@gmail.com (A.A.); fioretti@unina.it (A.F.); menna@unina.it (L.F.M.); 2 Department of Electrical Engineering and Information Technology, Federico II University of Naples, 80125 Naples, Italy

**Keywords:** animal-assisted interventions, co-therapist dogs, zoonosis, patients, risk factors, contact

## Abstract

Animal-assisted therapy (AAT) is a non-pharmacological therapy aimed at people with physical and/or mental disabilities. Therefore, it is necessary to carry out interventions that guarantee its benefits for patients while also avoiding the risk of zoonoses due to contact with the animals or their mucous membranes. The present study aimed to detect the occurrence of *Pasteurella multocida* in the oral cavity of dogs attending a “dog educational centre” and training for AAT interventions. In addition, some of the potential predictable factors of infection (i.e., age, sex, breed, and living conditions) were analyzed. In total, 25/200 dogs examined (12.5%; 95% confidence interval = 8.4–18.1%) were positive for *P. multocida*, as confirmed by PCR. Sex, breed, and living conditions were risk factors associated with *P. multocida* as revealed by the logistic regression analysis. Specifically, cross-bred female dogs living prevalently outdoors were significantly associated with the presence of *P. multocida* (*p* < 0.05). This study represents the first epidemiological survey of the prevalence of *P. multocida* in the oral cavity of dogs involved subsequently in AAT interventions, highlighting the potential risk of *P. multocida* infection in patients, often belonging to risk categories (e.g., children, the elderly, and immunocompromised individuals). Therefore, healthcare guidelines could be suggested to integrate the current literature related to the health check of dogs involved in AAT. In this way, it could be ensured that, even with bodily contact during AAT, the risk of pathogen transmission by the co-therapist dog can be avoided.

## 1. Introduction

The human–animal bond is considered as “a mutually beneficial and dynamic relationship between people and animals that is influenced by behaviors that are essential to the health and well-being of both” [1]. Likewise, the connection with other living organisms (i.e., biophilia) is considered a fundamental biological human need [1]. As is known, dogs generously contribute to many aspects of medicine and their relationship with humans is becoming ever closer, more frequent, and important—particularly concerning physical as well as psychological benefits [2,3,4]. In this regard, dogs also play a central role in animal-assisted therapy (AAT) as co-therapists or as a support for people with physical or mental health problems. Animal-assisted therapy is a goal-oriented, planned, and structured therapeutic intervention which is directed and/or delivered by health, education, and human service professionals (e.g., psychologists) and focuses on the socio-emotional functioning of the patients, either in a group or individual setting [5]. Usually, these therapeutic interventions are performed in healthcare facilities and are addressed to patients with mental or physical distress, depression, dementia, autism, or other illnesses [6,7,8]. During AAT, patients (usually young, old, or immunocompromised people) interact with dogs through different activities of an interspecific nature (i.e., petting, brushing, leading on a leash, hiding a ball, etc.) [4]. In this regard, Shen et al. [9] showed that bodily contact significantly influenced the effectiveness of AAT. On the other hand, during these interventions, because of repeated contact with the dog’s mucosae (e.g., mouth mucosa), patients can be exposed to zoonotic pathogens (e.g., bacteria, viruses, and fungi) transmitted by the dog through direct contact [3,10,11,12,13,14,15,16,17]. Several reports describe human *P. multocida* infections acquired subsequent to close contact with a dog, such as sharing a bed or by licking or sniffing [10,16,18]. *P. multocida* is part of the normal mouth microbiota of mammals; in dogs, its oropharyngeal colonization rates range from 50% to 66% [15]. Zoonotic transmission usually occurs through a bite from a dog and by contact with the animal’s saliva or nasal secretions [19,20,21]. Although cases of *P. multocida* infection have been reported more often in people from risk categories, such as young children, the elderly, pregnant women, or immunocompromised individuals [22], case reports of infection have also been reported in immunocompetent subjects [23,24,25,26]. In humans, *P. multocida* can cause serious infections, both invasive and localized, in the oral cavity, respiratory tract, and soft tissue, including pharyngitis, sinusitis, meningitis, tracheobronchitis, pneumonia, empyema, and abscess [10,18,23,24,25,26,27,28,29,30].

Considering that bodily contact is the main way to ensure the efficacy of AAT, with the high prevalence of *P. multocida* in the mouth microbiome of dogs and the possibility of transmission during the interaction time in the setting, the present study was carried out in order to evaluate the prevalence of *P. multocida* in the oral cavities of dogs in training for future interventions of AAT; in addition, the study aimed to evaluate whether age, sex, breed, and living conditions may represent predictable factors for the presence of this bacterium in the oral cavity of the dog.

Our evidence could be useful to suggest new guidelines regarding health protocols of dogs involved in AAT by integrating the information from current literature. In this way, bodily contact and other activities with dogs during AAT may be ensured while avoiding the risk of pathogens, such as *P. multocida*, being transmitted.

## 2. Materials and Methods

### 2.1. Sampling

The present study was performed from May to November 2018 on 250 dogs attending a dog educational centre in Southern Italy. As a preliminary step, the owners were interviewed using a pretested, standardized questionnaire to obtain a full history for each dog. The questionnaire was divided in two main parts: the first part included details on the animal’s age, sex, breed, and living conditions, whereas the second concerned the medical history of the dog (Supplementary Materials).

A homogeneous sample of 200 healthy and not neutered dogs (based on the second part of questionnaire) was classified by several categories: two age groups, one representing animals ranging from three to twelve months old (n = 30) and the other representing animals over twelve months (n = 170); two sex groups—male (n = 125) and female (n = 75); two breed groups—crossbred (n = 110) and purebred (n = 90); two living conditions groups—indoor (n = 142) and outdoor (n = 58). Each dog was individually sampled using sterile, cotton-tipped swabs in the oral cavity (palate, internal gums and teeth, and tongue). All of the sampled dogs showed an absence of a periodontal procedure.

The animals examined in this study were sampled upon approval by the Animal Ethics and Welfare Committee of the University of Naples Federico II (protocol number 10418/03 February 2014).

### 2.2. Isolation and Characterization of P. multocida

Oral swab samples were collected from the animals and immediately inoculated onto blood agar supplemented with 2 μg/mL clindamycin, and then incubated aerobically at 37 °C for 24 h. All oxidase-positive, catalase-positive, and small Gram-negative rods or coccobacilli observed under the light microscope were sub-cultured. The identification of *P. multocida* colonies was first performed biochemically [31,32], and then confirmed colonies were picked for DNA extraction using the PrepMan sample reagent (PE Applied Biosystems), following the manufacturer’s protocol. For *P. multocida* PCR identification, a pair of primers (Eurofins) were used: KMTJB-for TGCCACTTGAAATGGGAAATG and KMTJB-rev AATAACGTCCAATCAGTTGCG, (available in GenBank) encoding the outer-membrane protein (KMT-1) [33]. The reaction mixture (50 μL) contained 2 μL of mix oligos, 25 μL of MyTaq Red Mix (Bioline), and 2 μL of DNA. In total, forty PCR cycles of denaturation at 95 °C for 1min, annealing at 57 °C for 15 s, and elongation at 72 °C for 10 min were performed in a Model 9600 thermal cycler (PE Applied Biosystems). The PCR products were separated by electrophoresis on 1.8% agarose gel (Gibco-BRL), stained with ethidium bromide, and visualized under UV light, and the results were recorded using a ChemiImager 5500 (BSI). PCR amplified without DNA was used as a negative control, whereas reference *P. multocida* strains ATCC 43137 were used as positive controls (LGC Promochem).

### 2.3. Statistical Analyses

The chi-square test was used as a statistical method to determine whether age, sex, breed and living conditions were predisposing factors for *P. multocida* positivity. Furthermore, the Cochran–Mantel–Haenszel test was used for the independent variables that showed significance (*p* < 0.05) as well as to obtain an adjusted odds ratio (OD) with a 95% confidence interval (CI). Statistical analyses were performed in R [34,35]. Moreover, to gain insight into the different factor combinations affecting positivity for *P. multocida*, we performed a logistic multivariate regression [36] over all of the factors (age, sex, breed, and living conditions) in order to identify significant predisposing factor combinations with the following formula:

Log [p/(1 − p)] = β0 + β1 × sex + β2 × breed + β3 × living conditions + β4 × age(1)

where p is the probability to be positive for *P. multocida*; β_i are the regression coefficients; coding of the factors was the following: female = 0, male = 1; cross-breed = 0, pure-breed = 1; indoor = 0, outdoor = 1; young = 0, old = 1. This analysis was carried out in R [34,35].

## 3. Results

Out of the 200 dogs examined, 25 subjects (12.5%; 95% confidence interval (CI) = 8.4–18.1%) were positive for *P. multocida*, as found by the biochemical identification and then confirmed by PCR. As shown in Table 1, female dogs showed a high prevalence for *P. multocida* of 22.7%, whereas male dogs showed a prevalence of 6.4%. This difference was statistically significant (*p* < 0.05). Moreover, crossbred dogs showed a prevalence of 18.8% (95% CI = 14.0–23.5%) for *P. multocida*, whereas purebred dogs showed a prevalence of 5.6% (95% CI = 3.0–9.5%); this difference was statistically significant (*p* < 0.05). Finally, dogs living outdoor showed a prevalence of 22.4% (95% CI = 6.5–11.0%) for *P. multocida*, whereas dogs living indoors showed a prevalence of 8.4% (95% CI = 15.0–31.5%); this difference was statistically significant (*p* < 0.05). In contrast, there was no significant difference related to age (*p* > 0.05). With respect to the Cochran–Mantel–Haenszel test results, sex and living conditions were potential predictable risk factors for *P. multocida* positivity (Table 2).

Regarding the logistic regression analysis, the results confirmed that age was not a predictable risk factor (old dogs: 21/170 = 0.12; young dogs: 4/30 = 0.13, *p* > 0.05). On the contrary, sex, breed, and living condition factors showed significant predictable risk values as reported in the corresponding computed coefficients and *p*-values in Table 3 and Table 4. It should be highlighted, for example, that habitat is the most important factor because outdoor living can almost triplicate the OR (associated term in Table 3 is 2.870). In summary, *P. multocida* positivity matched with high probability in female, cross-bred, outdoor dogs (*p* < 0.05). 

Coefficients β_i in the first row are not directly interpretable, therefore, in the third row, we reported the corresponding odds ratio (OR):

[exp(β_i) = p/(1 − p)](2)

Specifically, exp(β_0) is the OR corresponding to the factor combination of female/cross-bred/indoor (see first row in Table 4); when considering other factor combinations, the term exp(β_0) must be multiplied by the exp(β_i) if the i-th factor is coded with 1 (e.g., when considering male/cross-bred/indoor (see second row in Table 4), the OR is given by exp(β0) * exp(β1) because male is coded with 1).

Probabilities are estimated by means of counts and number of actual positives clustered with the expected number of positives.

## 4. Discussion

The present study represents the first evaluation of the presence of *P. multocida* in the oral cavity of dogs being trained for AAT, in order to avoid the risk of zoonotic transmission to patients involved in this non-pharmacological therapy. The findings of the present study showed that *P. multocida* was isolated in 25/200 dogs attending a dog educational center. Particularly, sex (female vs. male) and living conditions (outdoor vs. indoor) were significant risk factors for *P. multocida* positivity as shown in Table 2. According to the results of our logistic regression analysis (reported in Table 3 and Table 4), the match of female/cross-bred/outdoor dogs showed high probability in the epidemiology of *P. multocida* infections. In our opinion, higher positivity in female dogs (22.7%), was not clearly understood considering the lack of data present in the literature. We can only hypothesize that the periodic change in the physiology of the reproductive apparatus of females and the different behavioral set with respect to males could be predisposing factors to be further explored. Regarding the greater positivity of crossbreed dogs (18.8%), it is not possible to give a motivation that has scientific support. The higher prevalence of *P. multocida* in dogs living outdoors (22.4%), might be related to the possible repeat contact with excreta of other vector animals (i.e., birds, rodents, or wild mammals) [37]. Contrasting data concerning *P. multocida* prevalence in the oral cavity of dogs involved in animal-assisted interventions were reported in the literature. Guay D.R. [15] reported the prevalence of *P. multocida* in the oral cavity of dogs, with a range from 50% to 66%. Meanwhile, Lefebvre et al. [16] showed the presence of 7/102 (6.9%) positive samples in dogs visiting hospitalized people in Ontario. Although it is difficult to speculate on these data, we can only hypothesize that different factors, such as geographic area, recent changes in the eating and hygiene habits of dogs, the method of sampling, and methods of bacterial identification, may have influenced the different prevalence of each study. In contrast, our study represents the first evaluation of *P. multocida* risk infection in dogs being trained as co-therapists in AAT. In fact, this non-pharmacological therapy has been performed in healthcare settings such as hospitals or healthcare facilities and is often aimed at patients belonging to risk categories (e.g., dialysis patients, Alzheimer’s disease patients, and immunosuppressed or immunocompromised patients) [7,38,39,40]. In line with the current literature, the zoonotic risk is much higher for immunocompromised people but is also present for immunocompetent people [23,24,25,41]. In recent scientific literature, different case reports of transmission to humans through licking and other forms of contact with dogs have been reported. Ryan and Feder [27] reported a case of meningitis from *P. multocida* in a 12-day-old child due to frequent licking by house dogs. The authors point out that contact with a dog’s saliva by licking can result in exposure to commensal organisms from its oral cavity, such as *P. multocida* or other bacteria. Navarro-Navajas et al. [21] described a clinical case of *P. multocida* bacteremia in an 88-year-old male patient who became infected by contact with a dog’s saliva, with whom he shared food. The authors suggested maintaining a high degree of attention especially for immunocompromised patients when they are in contact with pets. Zarlasht and Khan [26] presented a case of *P. multocida* bacteremia that occurred following contact with a dog’s saliva in a 61-year-old immunocompetent male subject. Therefore, the authors recommended considering this organism as a differential diagnosis not only in immunocompromised patients, but also in immunocompetent patients. Maraki et al. [25] described a case of a *P. multocida* infection in a 57-year-old immunocompetent male adult who had a decubitus ulcer of the lower limbs, emphasizing the need to avoid contact between the wound and the saliva of dogs. Abreu et al. [28] reported the case of a urinary tract infection by *P. multocida* in an 83-year-old male person. In addition, they performed a molecular genetic analysis of the *P. multocida* strains obtained by the patient and his dog, strongly suggesting a zoonotic transmission of this bacterium. Bardou et al. [24] reported a case of *P. multocida* meningitis in a 25-year-old immunocompetent woman through contact with her dog’s saliva. Although the reported cases of infection are all individual, of the *P. multocida* infections, we underlined the variety of infected people (i.e., age, gender, immune status) and their respective clinical conditions.

Even in AAT, the patients involved have very different characteristics both in terms of age and, above all, in the type of disease (e.g., Alzheimer’s, autism, depression, ADHD, and others).

Considering the few data and more generic guidelines often referenced in the scientific literature, our findings might serve to enrich the general recommendations for the health control of dogs and related risk assessment in the field of AAT [42,43,44,45]. In addition, given the prevalence of *P. multocida* found in our study (12.5%) and considering the close and necessary contact between co-therapist dogs and patients, we recommend applying some simple rules of hygiene and behavior: perform a microbiological analysis approach of the oral cavity in all animals involved in AAT as a sane screening procedure to preserve the health of patients; keep the dog indoors by avoiding uncontrolled external access, especially in the days preceding the AAT sessions; disinfect the dog’s coat and especially the mouth and nostrils (through wipes or patented preparations for the dog); pay attention to the game equipment and tools that are brought into the setting; avoid contact with other dogs or other animal species whose clinical history is unknown.

### Limits

Our epidemiological investigation has some weaknesses since it only addressed *P. multocida* presence when there are other bacterial species of commensal zoonotic agents in the oral cavities of dogs (i.e., *Capnocitophaga canimorsus*); the number of dogs sampled was not very large and, at the time of sampling, the subjects were still in training or being selected; only four predictable infection factors (i.e., age, sex, breed, and living conditions) were considered; antibiotic sensitivity tests of isolated strains were not performed; the sampling was not repeated despite suggestions and corrections on lifestyle habits to limit the possibility of infection from the environment.

## 5. Conclusions

The dog represents the species most frequently involved in Animal-assisted Interventions (AAIs) and, particularly, in AAT. Knowing that its health status is an essential way to protect both the health of patients and the well-being of the animal itself, specific health checks targeting zoonotic pathogens, which are sometimes underestimated, should be standardized and become routine for co-therapist dogs who must interact with patients or people in a healthcare setting. In addition, it is important to detect *P. multocida* infections as they represent a latent risk in the case of contact with patients who are co-infected during AAT, considering that serious and systemic infections are not only determined by bites and scratches but also by contact with animal secretions, which are a potential transmission source.

Therefore, by our findings, dogs that are considered to be at high risk of *P. multocida* infection (i.e., female, crossbred, outdoor housed) should be screened for infection as a priority alongside the other hygiene measures listed above.

Obviously, our evidence is not intended to represent a deterrent to carrying out AAT with dogs but an invitation to work with them carefully, allowing interaction with the animal while respecting the safety of the setting. Therefore, clear general hygiene rules should be reported and strictly applied, particularly in the setting of AAT.

## Figures and Tables

**Table 1:** Detailed sampling data of the dogs (age, sex, breed, and living conditions) and related positivity to *P. multocida*.

Dogs Data	No. of Samples	No. of Positive Samples (%)	CI ^†^ (95%)	*p* *
Age				
3–12 months	30	4 (13.33)	6.0–25.5	>0.05
>12 months	170	21 (12.35)	11.30–14.5	
Sex				
Female	75	17 (22.7)	16.5–30.5	0.001
Male	125	8 (6.4)	4.5–9.5	
Breed				
Crossbred	110	20 (18.8)	14.0–23.5	0.01
Purebred	90	5 (5.6)	3.0–9.5	
Living conditions				
Outdoor	58	13 (22.4)	15.0–31.5	0.01
Indoor	142	12 (8.4)	6.5–11.0	

^†^ CI 95%: Confidence interval (95%). * *p*: χ^2^.

**Table 2:** Cochran–Mantel–Haenszel test.

Independent Variable	Standard Error	*p* Value	Odds Ratio	95% Confidence Interval	
				Low	High
Sex					
Female vs. Male	0.45	0.001	4.28	1.74	10.51
Living conditions					
Outdoor vs. indoor	0.43	0.01	3.13	1.33	7.35

Dependent variable is *P. multocida* positivity.

**Table 3:** Logistic regression coefficients and corresponding *p*-values.

	β0	β1 (Sex)	β2 (Breed)	β3 (Living Conditions)
coefficient	−1.151	−1.496	−1.382	1.054
*p*	0.003	0.002	0.01	0.02
exp(coefficient)	0.316	0.224	0.251	2.870

**Table 4:** Height factor combinations and corresponding odds ratio (OR) and probabilities (normalized to interval 0–1) predicted with the logistic model.

Sex	Breed *	Living Conditions	Predicted Probability	Estimated Probability (CI) **	Count	Actual Positives	Expected Positives	OR ^†^
F	C	Indoor	0.240	0.230 (0.090, 0.436)	26	6	6.245	0.316
M	C	Indoor	0.066	0.061 (0.013, 0.169)	49	3	3.240	0.071
F	P	Indoor	0.073	0.125 (0.027, 0.324)	24	3	1.763	0.079
M	P	Indoor	0.017	0 (0.000, 0.082)	43	0	0.751	0.018
F	C	Outdoor	0.476	0.400 (0.163, 0.677)	15	6	7.136	0.907
M	C	Outdoor	0.169	0.250 (0.087, 0.491)	20	5	3.378	0.203
F	P	Outdoor	0.185	0.200 (0.025, 0.556)	10	2	1.855	0.228
M	P	Outdoor	0.048	0 (0.000, 0.247)	13	0	0.631	0.051
Total					200	25		

* C: crossbred; P: purebred; ** Confidence interval; ^†^ Odds ratio.

## Supplementary Materials

The following are available online at https://www.mdpi.com/1660-4601/17/17/6385/s1, Health questionnaire and life habits of the dogs.
