# Supplementary material for: Occurrence of Pasteurella multocida in Dogs Being Trained for Animal-Assisted Therapy
Source: Int J Environ Res Public Health. 2020 Sep 2;17(17):6385. doi: 10.3390/ijerph17176385 (PMC7503519; doi:10.3390/ijerph17176385)
Supplement: Supplementary file 1 [file ijerph-17-06385-s001.pdf]

ijerph-890836 <Supplementary Materials>

Health questionnaire and life habits of the dogs

Owner's Name: ___________________________
Owner's e-mail ___________________________
Dog's Name _________________________

Please choose or indicates your responses:

Part 1 – Characteristics of the dog and life habits

Age of the Dog □ 3 - 12 months □ > 12 months
Sex of the Dog F □ M □ (neutered yes □ no □)
Dog Breed ________________________

1. Does your dog go outdoors or stay indoors?
indoor □
outdoor □

2. Does your dog ever go to dogs' gathering areas, public parks or any other places where dogs meet?
Yes □ No □

Part 2 – Medical history

3. Has your dog had a coprological examination for endoparasites in the last year?
Yes□ No □
If yes, please list kind of parasites found: ________________________
If yes, please list type of treatment used: ____________________________________

4. Have you found ectoparasites on your dog in the last year?
Yes □ No □
If yes, please list ectopesticides you used: ________________________

5. Have you found fungus and mites on your dog's skin in the last year?
Yes □ No □
If yes, please list kind of fungus and mites found: ________________________
If yes, please list treatment used: ____________________________________

6. Which vaccines does your pet usually receive?
- Rabies □
- Distemper □
- Parvo □
- Leptospirosis □
- Bordetella □
- CAV-1 (canine adenovirus type 1) □

7. Has your dog had an examination for *Erlichia* antibody titers in the last year?
Yes□ No □

8. Has your dog had an examination for *Leishmania* antibody titers in the last year?
Yes□ No □

9. Has your dog had a blood streak for Heartworm in the last year?
Yes□ No □

10 Please list/describe any previously diagnosed illnesses, chronic conditions, and past surgeries related to your dog:

____

____

11. Please describe any health concerns you may have at this time:

____

____

12. Please, indicates any recent or ongoing antibiotic treatment:

____

____

Date: __/__/____
